# Comparing culture and molecular methods for the identification of microorganisms involved in necrotizing soft tissue infections

**DOI:** 10.1186/s12879-016-1976-2

**Published:** 2016-11-08

**Authors:** Vibeke Børsholt Rudkjøbing, Trine Rolighed Thomsen, Yijuan Xu, Rachael Melton-Kreft, Azad Ahmed, Steffen Eickhardt, Thomas Bjarnsholt, Steen Seier Poulsen, Per Halkjær Nielsen, Joshua P. Earl, Garth D. Ehrlich, Claus Moser

**Affiliations:** 1 Center for Microbial Communities, Department of Chemistry and Bioscience, Aalborg University, Aalborg, Denmark; 2 Life Science Division, The Danish Technological Institute, Taastrup, Denmark; 3 Center for Genomic Sciences, Allegheny-Singer Research Institute, Pittsburgh, USA; 4 Department of Immunology and Microbiology, University of Copenhagen, Copenhagen, Denmark; 5 Department of Clinical Microbiology, Copenhagen University Hospital, Rigshospitalet, Denmark; 6 Department of Biomedical Sciences, University of Copenhagen, Copenhagen, Denmark; 7 Center for Genomic Sciences, Philadelphia, PA USA; 8 Departments of Microbiology and Immunology, Center for Advanced Microbial Processing, Institute for Molecular Medicine and Infectious Disease, Philadelphia, PA USA; 9 Departments of Microbiology and Immunology, Philadelphia, PA USA; 10 Otolaryngology-Head and Neck Surgery, Drexel University College of Medicine, Philadelphia, PA USA

**Keywords:** Necrotizing soft tissue infections, Microorganisms, 16S rRNA, Cloning, Direct Sanger sequencing, Ibis T5000 biosensor, 454 pyrosequencing, qPCR, FISH

## Abstract

**Background:**

Necrotizing soft tissue infections (NSTIs) are a group of infections affecting all soft tissues. NSTI involves necrosis of the afflicted tissue and is potentially life threatening due to major and rapid destruction of tissue, which often leads to septic shock and organ failure. The gold standard for identification of pathogens is culture; however molecular methods for identification of microorganisms may provide a more rapid result and may be able to identify additional microorganisms that are not detected by culture.

**Methods:**

In this study, tissue samples (*n* = 20) obtained after debridement of 10 patients with NSTI were analyzed by standard culture, fluorescence *in situ* hybridization (FISH) and multiple molecular methods. The molecular methods included analysis of microbial diversity by 1) direct 16S and D2LSU rRNA gene Microseq 2) construction of near full-length 16S rRNA gene clone libraries with subsequent Sanger sequencing for most samples, 3) the Ibis T5000 biosensor and 4) 454-based pyrosequencing. Furthermore, quantitative PCR (qPCR) was used to verify and determine the relative abundance of *Streptococcus pyogenes* in samples.

**Results:**

For 70 % of the surgical samples it was possible to identify microorganisms by culture. Some samples did not result in growth (presumably due to administration of antimicrobial therapy prior to sampling). The molecular methods identified microorganisms in 90 % of the samples, and frequently detected additional microorganisms when compared to culture. Although the molecular methods generally gave concordant results, our results indicate that Microseq may misidentify or overlook microorganisms that can be detected by other molecular methods.

Half of the patients were found to be infected with *S. pyogenes*, but several atypical findings were also made including infection by a) *Acinetobacter baumannii*, b) *Streptococcus pneumoniae*, and c) fungi, mycoplasma and *Fusobacterium necrophorum*.

**Conclusion:**

The study emphasizes that many pathogens can be involved in NSTIs, and that no specific “NSTI causing” combination of species exists. This means that clinicians should be prepared to diagnose and treat any combination of microbial pathogens. Some of the tested molecular methods offer a faster turnaround time combined with a high specificity, which makes supplemental use of such methods attractive for identification of microorganisms, especially for fulminant life-threatening infections such as NSTI.

## Background

The spectrum of diseases referred to as soft tissue infections is diverse. Their common characteristic is that they involve infection of the skin, subcutaneous tissue, fascia or muscle [1]. These infections range from common superficial epidermal infections to potentially life threatening cases of necrotizing soft tissue infections (NSTI) [2]. The incidence of NSTI has been estimated to be 4 cases per 100,000 person-years in the USA [3]; thus, an average practitioner will only see one or two cases during their career [4, 5], and may therefore not be sufficiently familiar with the disease to ensure a rapid diagnosis and appropriate treatment [5]. Treatment of NSTI involves immediate aggressive surgical debridement and administration of intravenous broad-spectrum antibiotics. Some centers also use systemic administration of non-specific immunoglobulin as well as hyperbaric oxygen treatment. Establishing the diagnosis can be a challenge in managing NSTI, because the early signs are non-specific and include local erythema and swelling with warmth and pain out of proportion to physical findings [5, 6]. As the disease progresses, bullae filled with serous fluid are formed, and eventually large hemorrhagic bullae, skin necrosis, fluctuance, crepitus as well as sensory and motor deficits become apparent [2, 6]. Despite many advances in the understanding of NSTI and great improvements in medical care, the mortality associated with NSTI remains high [2, 5]. Different mortality rates have been reported, but are generally in the range of 16-24 % [4, 6, 7].

The etiology of necrotizing fasciitis is variable and not fully understood. In some cases an antecedent penetrating injury is present (such as skin trauma, varicella, or burns) [6, 8, 9]. The skin trauma may be caused by surgery or may even be caused by a trivial event such as an insect bite, scratch, or abrasion [10, 11]. In many cases however, no identifiable cause can be found [6, 8–10, 12]. In these cases it is hypothesized that necrotizing fasciitis may result from hematogeneous seeding from a reservoir in the oropharynx or other anatomic site [9, 13]. Most patients who develop necrotizing fasciitis have pre-existing conditions that render them susceptible to infection, including diabetes mellitus, advanced age, immune suppression, peripheral vascular disease, obesity, smoking, drug and alcohol misuse [4–6, 11, 14]. The necrotizing changes associated with NSTI lead to devitalization of the infected tissue, which provides a suitable environment for further microbial growth, setting the stage for major and rapid destruction of tissue [1, 2]. Infection can spread as fast as 1 in. per hour with little overlying skin change [5]. It is hypothesized that rapid tissue destruction and severe pain associated with NSTI is caused by the interaction of microorganisms and their toxins with the human coagulation system, leading to hypercoagulation, vascular occlusion and microvascular thrombosis as well as direct triggering of the nerves has been suggested recently [15, 16]. The resulting poor tissue perfusion also has implications on the treatment strategy, since the antibiotic concentration at the infection site may be insufficient [12].

Historically, NSTI has been classified into specific types based on anatomic location or microbial findings. However, it has been suggested that such classifications lead to undue complication of the issue. It is argued that the most important information to be established is the presence of a necrotizing component, distinguishing NSTI from a milder condition such as cellulitis that should respond to antibiotics alone [2, 4, 10, 11, 17]. On the other hand, the correct identification of involved microorganisms has important implications on the antibiotic treatment since *S. pyogenes* and *Clostridium perfringens* need different treatment modalities [2, 18, 19] than, for example, methicillin-resistant *Staphylococcus aureus* [20], or *Streptococcus pneumoniae* [21]. In addition, accurate microbial diagnosis is pivotal for identifying the primary microbial entry site or focus of the infection, which is also of substantial importance for optimal handling of the NSTI. The multiple microbial etiologies of NSTI support the empiric use of broad-spectrum antibiotics in high doses until accurate microbial diagnosis has been obtained.

The microbial communities involved in NSTI have previously been investigated by culture-dependent methods. However, it is possible that additional microorganisms, which may not be detectable by standard cultural methods, are involved in the infections. Recent studies of numerous infectious conditions using molecular diagnostics have revealed that many of what were once thought to be monomicrobial infections are in fact polymicrobial, although the significance of the additional findings is not always completely understood [22–25]. Presently, various molecular methods are available that may be able to identify additional microorganisms and offer a more rapid identification than routine culture-based methods. Because of the rapid progression of the disease it is of paramount importance that the etiologic pathogens be rapidly and accurately identified. The initial empiric antimicrobial treatment can be modified in cases of rare or surprising microbial findings to target the involved microorganisms to minimize extensive and/or life-threatening damage to the patients. In other cases fast and accurate diagnoses can be important for the clinician to support the relevance of the antibiotic treatment initiated and to prevent unnecessary and sometimes inadequate antibiotic treatments in these critically ill patients.

In this study, we investigated several molecular methods for identification of microorganisms, including the Ibis T5000 biosensor, quantitative polymerase chain reaction (qPCR), and 16S rDNA and D2LSU gene analysis by direct sequencing, near full length 16S rRNA clone libraries including sequencing and 454 pyrosequencing. These findings were then compared to those of routine cultural methods.

## Methods

### Patients and samples

Samples in this study were obtained from NSTI patients by debridement of the infected area, performed at Rigshospitalet (Copenhagen, Denmark). A total of 20 samples from 10 patients were included (Table 1). Disposing factors included diabetes mellitus, adiposity and chronic leg ulcur, inguinal hernia, leukemia, immunosuppression. Limb defects and burn wounds. Several patients experienced severe sepsis or septic shock including organ failure. The debrided tissues were immediately transported to the Department of Clinical Microbiology where each sample was divided into three aliquots for standard culture experiments, molecular analyses, and PNA-FISH experiments. The samples for molecular analyses and the FISH experiments were transferred to tubes containing glycerol or ethanol respectively, and kept frozen until analysis. In addition, other culture results were checked for in the patient’s files (e.g. positive blood cultures).

**Table 1:** Patient information and number (n) of surgical samples investigated by molecular methods in this study

Patient	Anatomical site	Age range	Antibiotics	Outcome
1 (*n* = 4)	Femur	70–79	meropenem, ciprofloxacin and metronidazole (supplemented with clindamycine after sampling).	Survival.
2 (*n* = 2)	Crus	20–29	meropenem, ciprofloxacin and clindamycine.	Survival.
3 (*n* = 1)	Crus	60–69	meropenem, ciprofloxacin and clindamycine. (Cefuroxime and Gentamicin before transferral)	Death within 24 h.
4 (*n* = 2)	Arm	60–69	meropenem, ciprofloxacin and clindamycine.	Survival.
5 (*n* = 1)	Inguina	60–69	meropenem, ciprofloxacin and clindamycine (suppl. metronidazole after sampling).	Survival.
6 (*n* = 1)	Vulva	60–69	meropenem, ciprofloxacin and clindamycine.	Death due to disposing disease.
7 (*n* = 2)	Neck	70–79	PEN and metronidazole. After recurrence: meropenem, metronidazole, linesolid and moxifloxacin.	Survival.
8 (*n* = 3)	Shoulder	60–69	meropenem, ciprofloxacin and clindamycine.	Survival.
9 (*n* = 2)	Shoulder	40–49	meropenem, ciprofloxacin and clindamycine.	Survival.
10 (*n* = 2)	Arm	50–59	meropenem, ciprofloxacin, clindamycine, metronidazole	Survival.

Antibiotics *abbreviations*: *MEPM* meropenem, *CPFX* ciprofloxacin, *MNZ* metronidazol, *CLDM* clindamycin, *PEN* penicillin

### Ethics

In all cases the material for molecular diagnostics was leftover debridement material from treatment and diagnosing the patients, which would have been discarded otherwise. No extra sampling from the patients was performed. In addition, patient files were only checked for the purpose of treating the patients and correlating microbial findings to the clinical findings. Data from the patient history is exclusively from internal notes in the Department of Clinical Microbiology for that purpose. Samples sent for further analysis were completely anonymized except for the principal investigator (CM). Samples have been destroyed after the study. Therefore, the present study is considered as quality assessment investigating the potential contribution of novel molecular techniques. Based on this, a written informed consent and ethics committee approval were not needed and Danish law was strictly complied.

### Culture

All culture analyses of the debrided afflicted area were performed at the Department of Clinical Microbiology at Rigshospitalet. All biopsies were analyzed by Gram-staining and culture. Both aerobic and anaerobic conditions were used. Biopsies were plated on brain heart infusion agar (BHIA, Statens Serum Institut (SSI), Copenhagen Denmark), coagulated agar, and 5 % horse blood agar (SSI) for cultures in 5 % CO_2_ atmosphere. Aerobic conditions included plating on modified Conradi-Drigalski (“Blue plates”, SSI), in serum bouillon, in thioglycollate media, and on tellurite agar (SSI) in a normal atmosphere. Colonies were further identified by use of Matrix-assisted laser desorption-ionization time of flight mass spectroscopy (MALDI-TOF MS), (Bruker, Bremen, Germany). Antibiotic resistance patterns were analyzed by disc diffusion test on blood agar (SSI) using Neosensitabs (Rosco Diagnostica, Taastrup, Denmark).

### DNA extraction

DNA was extracted from samples as described previously [26]. Briefly, the tissue samples were cut into small pieces under sterile conditions. Approximately 1 mm^3^ of tissue was transferred to a microcentrifuge tube containing tissue lysis buffer (ATL, Qiagen) and 20 mg/mL proteinase K (Qiagen). The sample was incubated at 56 °C until visual inspection indicated that lysis was achieved. 100 μL Zirconia/silica beads mixture (50 μL of 0.1 mm diameter, Biospec, PN: 11079101z and 50 μL of 0.7 mm diameter, Biospec, PN:11079107zx) was added to the microcentrifuge tube and the sample was homogenized for 10 min at 25 Hz using a Qiagen Tissuelyser (Model MM300, cat# 85210). DNA from the lysed samples was extracted using the Qiagen DNeasy Tissue kit, according to the manufacturer’s protocol. The DNA was eluted in 200 μL AE buffer (10 mM Tris · Cl; 0.5 mM EDTA, pH 9.0).

### Identification using MicroSeq® microbial Identification System

PCR was performed with primers that targeted the first 500 bases of the bacterial 16S rRNA gene or the D2LSU region of the fungal 28S rRNA gene. The resulting PCR products were sequenced using the MicroSeq® 500 kit (Applied Biosystems, Carlsbad, California) according to the manufacturer’s guidelines. The resulting DNA sequences were compared to the sequence library included in the MicroSeq® ID analysis software. In cases where sequencing resulted in mixed chromatograms due to 16S rRNA gene products from multiple species, these chromatograms were analyzed using RipSeq Mixed at www.ribseq.com.

### Construction and analysis of clone libraries

Clone libraries were constructed for all samples except 4A, 6A, 7A, 8A, 8B and 8C (due to insufficient volumes of DNA extract). The libraries consisted of near full length 16S rRNA genes (*E. coli* position 26–1390) which were obtained as described previously [27, 28]. Briefly, PCR amplicons were cloned using the TOPO TA Cloning ® kit (Invitrogen) according to the manufacturer’s instructions. For each surgical sample, 48 clones were subjected to plasmid purification and sequencing were performed by Macrogen Inc. (Korea) using M13F primer (and M13R primer in some cases). Manual refinement of sequences and construction of consensus sequences were done in CLC Main Workbench (CLC bio, Aarhus, Denmark). Sequences were checked for chimeras using the Mallard software package [29], aligned using SINA Web Aligner [30] and imported into the ARB software package [31] for taxonomic lineage assignment, using the non-redundant (NR) SSU Ref database from SILVA Release 106 as reference database. Operational taxonomic units (OTUs) were constructed across all patient samples for clones having a sequence similarity of more than 97 % since these sequences are typically assigned to the same species. One clone from each OTU was sequenced with both M13F and M13R primers. The resulting consensus sequences and their closest relatives in the database were selected to construct phylogenetic trees using neighbor joining, maximum parsimony and maximum likelihood methods. The non-redundant, near full-length 16S rRNA gene sequences, representing each OTU obtained in this study, were deposited in GenBank under the accession numbers (KP114666-KP114679).

### 454 pyrosequencing

Four hundred fifty four-based pyrosequencing was performed largely as described previously [32]. Briefly, the bar-coded FLX-titanium amplicon pyrosequencing targeted the V1–V2 region of the 16S rRNA gene (using 27 F and 338R primers). The DNA fragments were amplified using Platinum Hi-Fi taq polymerase (Invitrogen) with 800 μM dNTP, 2 mM MgCl_2_ and 400 nM of each primer. To each reaction 5 μL of template DNA was added and the volume was adjusted to 50 μL. The PCR incubation conditions were 94 °C for 2 min followed by 30 cycles of 94 °C for 30 s, 55 °C for 30 s, and 68 °C for 60 s, with a final extension at 72 °C for 7 min. Tag-encoded FLX amplicon pyrosequencing analyses utilized the Roche 454 FLX instrument with titanium reagents and titanium procedures (Roche).

Analysis of 16S rRNA gene amplicon sequences was performed using Quantitative Insights Into Microbial Ecology (QIIME v.1.3.0) pipeline [33]. The sequencing data was processed initially with AmpliconNoise [34] to remove noise. Then the QIIME pipeline separated the sequences into individual specimen communities based on the unique 5’ barcode sequence and utilized a suite of external programs to make taxonomic assignments and estimate phylogenetic diversity. These data were used to generate taxonomic summaries. The default settings in QIIME were employed for analysis, except that the sequences were grouped into operational taxonomic units using 99 % sequence similarity for clustering; taxonomic assignments were done using Greengenes taxonomy [35].

### Ibis T5000 assay

An aliquot of each DNA extract was loaded into each of 16 wells of an Ibis 96-well BAC (bacteria, antibiotic resistance genes, candida) detection plates (Abbott Molecular) and processed as described previously [36]. Briefly, PCR amplifications were carried out, and the resulting PCR products were then desalted in a 96-well format and sequentially electrosprayed into the TOF MS as described by the manufacturer. The spectral signals were processed to determine the mass of each strand of the PCR products, which in turn were used to derive the base compositions that were then compared to the Ibis database to obtain species level determinations for all microorganisms [37].

### Quantitative PCR

Quantification of *S. pyogenes* [38] and 16S rRNA genes [39] was performed using hydrolysis probe chemistry. The *S. pyogenes* assay is commercially available from Biosearch Technologies (Novato, CA). For each sample duplicate 25 μL reactions were run, each containing: 12.5 μL Brilliant® qPCR Master mix (Agilent Technologies, Santa Clara, California), 25 μg BSA (Sigma-Aldrich, Brøndby, Denmark), appropriate concentration of primers and TaqMan® probes (*S. pyogenes*: 400 nM primers and 100 nM probe, 16S rRNA: 900 nM primers and 200 nM probe), 0.75 μM ROX reference dye (Agilent Technologies) and 2 μL of template DNA. Measurements were obtained by absolute quantification using genomic DNA isolated from *S. pyogenes* (DSM 20565) and *P. aeruginosa* (DSM 1253) for total bacteria quantification. The number of isolated genomes was calculated based on DNA concentration (Quant-iT™ dsDNA Assay Kit (Invitrogen)) and genome size estimated to be 1.8 Mbp for *S. pyogene*s and 6.5 Mbp for *P. aeruginosa* (http://img.jgi.doe.gov/cgi-bin/pub/main.cgi). Dilution series of the genomic DNA covered a range of 10^6^-10^0^ genome copies. Reactions were run on an Mx3005P (Agilent Technologies) with the following program: 10 min at 95 °C, followed by 40 cycles of 30 s at 95 °C, 1 min at 60 °C.

### Analysis of quantitative data

The number of gene copies measured by qPCR was converted to number of CFU per gram sample using $$ \raisebox{1ex}{$\mathrm{C}\mathrm{F}\mathrm{U}/\mathrm{g}=\kern0.5em {\mathrm{C}}_{\mathrm{m}\mathrm{easured}}/{\mathrm{C}}_{\mathrm{genome}}*\left({\mathrm{V}}_{\mathrm{total}}/{\mathrm{V}}_{\mathrm{used}}\right)$}\!\left/ \!\raisebox{-1ex}{${\mathrm{m}}_{\mathrm{sample}}$}\right.. $$ Here C_measured_ is the number of copies measured and C_genome_ is the number of gene copies in the genome of one CFU. The standard deviation of all measurements above the detection limit of the assays was calculated. In cases where both the *S. pyogenes* and 16S rRNA assay were above the limits of detection, a two tailed *t*-test was used to provide a hypothesis test of the difference between population means. A statistical value of ≤ 0.05 was considered significant.

### Visualization of samples

The samples were prepared for visualization by imbedding in paraffin, which was sectioned (4 μm) and mounted on microscope slides and stored at room temperature. Before staining or hybridization, the slides were deparafinated by using 2x 5 min xylene, 2x 3 min 99.9 % EtOH, 2x 3 min 96 % EtOH, and washed 3x 3 min in sterile water. The deparafinated NSTI sections were analyzed by FISH using a mixture of Cy3-labeled “Strept probe” targeting *Streptococcaceae* [40] and a Cy5-labeled broad range bacteria probe (EUB-338) [41]. Hybridization was performed by covering the slide with 12 μL of hybridization buffer containing 0.9 M NaCl, 0.02 M Tris/HCl (pH 8), 0.01 % SDS, and 30 % formamide and probe mix (5 ng/μL of the respective probes) followed by incubation at 46 °C in a humid chamber. After 90 min. slides were rinsed with washing buffer (0.102 M NaCl, 0.02 M TRIS/HCl, 0.01 % SDS) preheated to 48 °C and then incubated in the washing buffer for 15 min at 48 °C. Subsequently, DNA of both bacteria and host cells was stained with 50 μg/ml 4’,6-diamidino-2-phenylindole (DAPI) and incubated for 15 min in darkness at RT after which the slides were washed with dH_2_0 and air dried. The slides were mounted with Vectashield (Vector labs) and a cover slip was added. Slides were investigated using a LSM 710 confocal laser scanning microscope (Zeiss, Germany).

## Results

### Identification of microorganisms by routine culture

The findings by routine culture at the Department of Clinical Microbiology, Rigshospitalet (Copenhagen, Denmark), mostly revealed the presence of one type of pathogenic bacteria in the surgical samples (in 7 of the 10 patients) (Table 2). These monomicrobial infections were primarily caused by streptococci (71 % of the monomicrobial infections), specifically *S. pyogenes*, *S. pneumoniae* and non-hemolytic streptococci. The remaining monomicrobial culture findings were identified as *Acinetobacter baumanii* (patient 3) and fungal infection (patient 7). Furthermore, two patients were found to harbor more than one microorganism (patients 5 and 6), where *Bacteroides fragilis* with *Clostridium paraputrificum* and *S. pyogenes* with *Escherichia coli* were found. One fourth of the samples investigated by culture did not result in growth of microorganisms. Three of these surgical samples originated from patients where other samples taken from the site of infection resulted in growth of microorganisms. The remaining culture-negative samples originated from a patient, where none of the samples resulted in growth of microorganisms (patient 10).

**Table 2:** Microorganisms detected by culture methods in surgical and other (often previous) samples from NSTI patients

	Other samples	Surgical samples		
Patient	Culture	Sample	Culture	Molecular methods
1	-	A	*Streptococcus pyogenes*	✓
		B	*Streptococcus pyogenes*	✓
		C	*Streptococcus pyogenes*	✓
		D	*Streptococcus pyogenes*	✓
2	*Streptococcus pyogenes* (blood culture)	A	*Streptococcus pyogenes*	✓
		B	*Streptococcus pyogenes*	✓
3	No growth	A	*Acinetobacter baumannii* (Gram positive cocci in chains by light microscopy)	✓^a^
4	*Streptococcus pyogenes* and CNS (Gram negative rods by light microscopy)	A	Non-hemolytic streptococci	✓
		B	No growth	^a^
5	-	A	*Bacteroides fragilis, Clostridium paraputrificum*	✓^a^
6	*Streptococcus pyogenes, Staphylococcus aureus* and *Enterobacteriaceae*	A	*Streptococcus pyogenes, Escherichia coli*	✓^a^
7	*Fusobacterium necrophorum*	A	Fungus	✓^a^
		B	No growth	^a^
8	*Streptococcus pneumoniae*	A	*Streptococcus pneumoniae*	✓
		B	*Streptococcus pneumoniae*	✓
		C	No growth	^a^
9	*Streptococcus pyogenes*	A	*Streptococcus pyogenes*	✓
		B	*Streptococcus pyogenes*	✓
10	*Staphylococcus aureus*	A	No growth	✓
		B	No growth	✓

In many cases the findings by culture were confirmed by the molecular methods (✓), or the molecular methods identified additional microorganisms (^a^). Text in brackets indicate relevant findings by light microscopy

-Indicates that no previous samples were taken for culture

### Identification of microorganisms by molecular methods

**Fig. 1:**
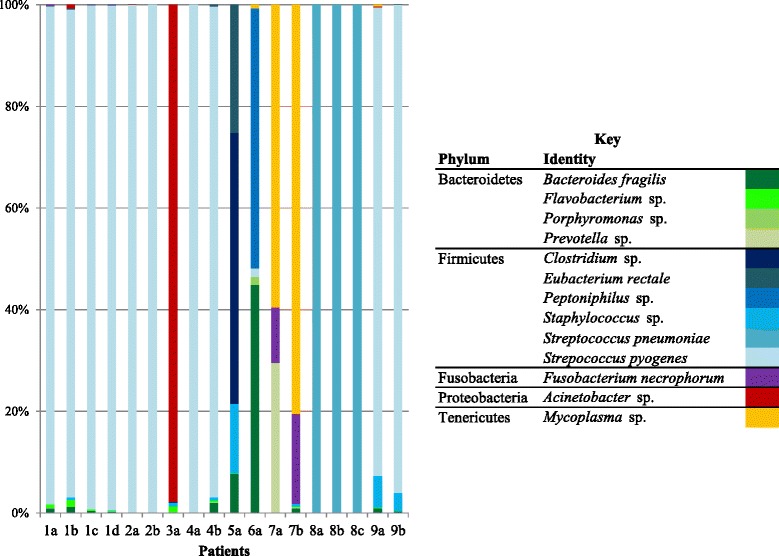
Taxa identified by pyrosequencing. The stacked graph illustrates the relative abundance of each taxon identified by pyrosequencing from the six samples (color coded according to the key)

Generally, the molecular methods confirmed the findings by culture (Table 2 and Table 3). However, using the multiple molecular methods, microorganisms were found in all samples including those that were culture-negative, and in most culture-positive cases additional microorganisms were identified by the molecular methods (Table 3, Fig. 1). Overall, the different molecular methods gave concordant results (although the16S rRNA clone libraries were only constructed for 16 of the samples). There were, however, cases where different microorganisms could only be detected by one molecular method. This was either due to misidentification or identification of additional species (Fig. 1). For patient 10, the results were difficult to interpret since the molecular methods gave differing results (*S. pyogenes* by Microseq, while Ibis found *S. pneumoniae*, *Clostridium septicum* and CoNS, and clone library and 454-pyrosequencing did not give results).

**Table 3:** Comparison of findings by molecular methods

Patient	Sample	Microseq	Sanger sequencing of clone libraries	Ibis T5000 biosensor	454-pyrosequencing
1	A	*Streptococcus pyogenes ✓*	*Streptococcus pyogenes ✓*	*Streptococcus pyogenes ✓*	*Streptococcus pyogenes ✓*
	B	*Streptococcus pyogenes ✓*	*Streptococcus pyogenes ✓*	*Streptococcus pyogenes ✓*	*Streptococcus pyogenes ✓*
	C	*Streptococcus pyogenes ✓*	*Streptococcus pyogenes ✓*	*Streptococcus pyogenes ✓*	*Streptococcus pyogenes ✓*
	D	*Streptococcus pyogenes ✓*	*Streptococcus pyogenes ✓*	*Streptococcus pyogenes ✓*	*Streptococcus pyogenes ✓*
2	A	*Streptococcus pyogenes ✓*	*Streptococcus pyogenes ✓*	*Streptococcus pyogenes ✓*	*Streptococcus pyogenes ✓*
	B	*Streptococcus pyogenes ✓*	*Streptococcus pyogenes ✓*	*Streptococcus pyogenes ✓*	*Streptococcus pyogenes ✓*
3	A	(*Streptococcus pyogenes*)	*Acinetobacter baumannii* ✓	*Acinetobacter baumannii ✓*	*Acinetobacter* sp.✓
4	A	*Streptococcus pyogenes ✓*	Not performed	*Streptococcus pyogenes ✓* *Streptococcus didelphis ✓*	*Streptococcus pyogenes ✓*
	B	*Streptococcus pyogenes*	*Streptococcus pyogenes*	*Streptococcus pyogenes*	*Streptococcus pyogenes*
5	A	(*Streptococcus pyogenes*)	*Clostridium paraputrificum* ✓ Uncultured bacterium	*Clostridium paraputrificum* ✓ *Bacteroides fragilis* ✓ (*Streptococcus agalactiae*)	*Clostridium* sp. ✓ *Bacteroides fragilis* ✓
6	A	*Streptococcus pyogenes ✓* (*Mycoplasma hominis*)	Not performed	*Streptococcus pyogenes ✓* *Escherichia coli ✓* *Bacteroides fragilis* (*Staphylococcus hominis*) (*Staphylococcus epidermidis*) (*Cladosporium cladosporioides*)	*Streptococcus pyogenes ✓* *Bacteroides fragilis*
7	A	*Mycoplasma* spp. *Fusobacterium necrophorum*	Not performed	*Mycoplasma* sp. *Fusobacterium necrophorum* *Candida albicans* ✓	*Mycoplasma* sp. *Fusobacterium necrophorum*
	B	*Mycoplasma salivarium*	*Mycoplasma salivarium* *Fusobacterium necrophorum*	*Mycoplasma* sp. *Fusobacterium necrophorum*	*Mycoplasma* sp. *Fusobacterium necrophorum*
8	A	*Streptococcus pneumoniae ✓*	Not performed	*Streptococcus pneumoniae ✓*	*Streptococcus pneumoniae ✓*
	B	*Streptococcus pneumoniae ✓*	Not performed	*Streptococcus pneumoniae ✓*	*Streptococcus pneumoniae ✓*
	C	*Streptococcus pneumoniae*	Not performed	*Streptococcus pneumoniae*	*Streptococcus pneumoniae*
9	A	*Streptococcus pyogenes*	*Streptococcus pyogenes*	*Streptococcus pyogenes*	*Streptococcus pyogenes*
	B	*Streptococcus pyogenes*	*Streptococcus pyogenes*	*Streptococcus pyogenes*	*Streptococcus pyogenes*
10	A	(*Streptococcus pyogenes*)	No PCR	(*Streptococcus pneumoniae*) (*Clostridium septicum*)	Low read count
	B	(*Streptococcus pyogenes*)	No PCR	(*Staphylococcus capitis/caprae*)	Low read count

Cases where the microorganisms were identified by culture are marked by ✓. For the 454-pyrosequencing only the concordant results are listed here (additional species are seen in Fig. 1)

() indicates that microorganism could only be found by one molecular method and not by culture

Of the molecular methods applied, only the Ibis T5000 biosensor could identify *Candida albicans* in sample 7B and *Cladosporium cladosporioides* in sample 6A.

### Verification of findings by qPCR and quantitative data

**Fig. 2:**
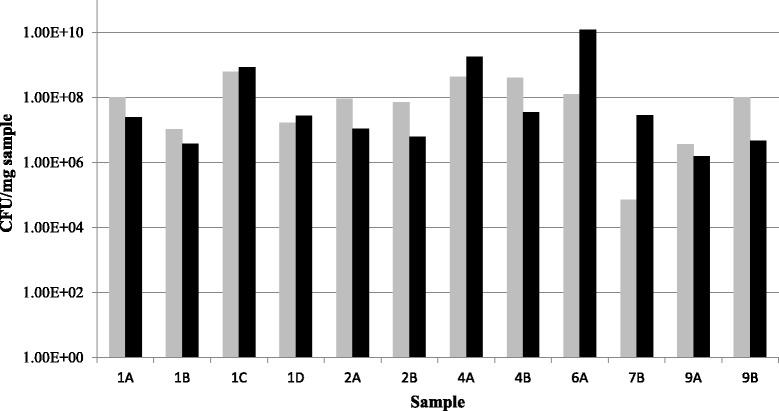
Relative abundance of *S. pyogenes*. Results by taqman qPCR for *S. pyogenes* (grey) and the 16S rRNA gene of all bacteria (black) given as CFU/mg sample. Only results where *S. pyogenes* were detected by qPCR are shown

The findings of *S. pyogenes* by molecular methods could generally be confirmed by qPCR (Fig. 2). Based on the measurements of bacterial 16S rRNA genes, *S. pyogenes* was the dominant microorganism in most of the samples, except 6A and 7B. This corresponds with the trend seen in the 454-pyrosequencing data (Fig. 1).

### Visualization of samples

**Fig. 3:**
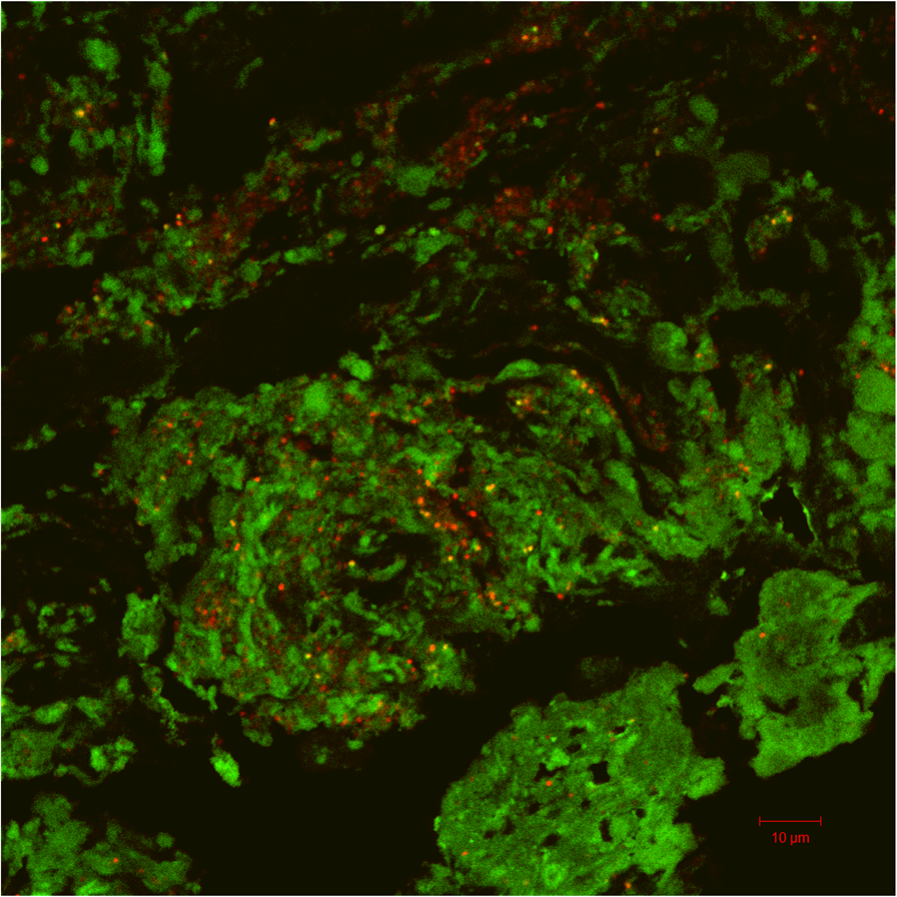
Visualization of NSTI samples obtained from patient 9. Images show *Streptococcaceae* (red), bacteria (green) and cells targeted by both Strept and EUB probe (yellow/orange). Background level for the EUB probe was intentionally set high to illustrate the structure of the debrided tissue. An area of sample 1A. Scale bar represents 10 μm

Using FISH it was possible to visualize bacteria in the NSTI samples. The bacteria observed were generally clustered together, and did not appear to penetrate into the lipid droplet of the fat cells, but were instead found in the matrix surrounding the fat cells (illustrated on the representative images obtained for samples from patient 1A, Fig. 3). In a few cases neither of the FISH probes resulted in visualization of cells, including samples 5A, 10A and 10B. Generally, the *Streptococcaceae*-specific FISH probe confirmed the cases where the bacteria were found by molecular methods.

## Discussion

NSTI is a serious, potentially lethal condition induced by microorganisms. The gold standard for identification of microorganisms involved in NSTI is culture and a number of studies have described microbiological findings during NSTI. However, newer molecular techniques hold the promise of providing additional information relating to the detection of unculturable organisms, with the added benefit of a shortened turnaround time. Therefore, the present study was designed to investigate the potential of adding molecular diagnostics to cultural studies in the diagnosis of NSTI. For the majority (15/20: 75 %) of samples included in this study, pathogens could be identified by culture (Table 2) with monomicrobial infections caused by *S. pyogenes* being the most frequent finding. The incidence of monomicrobial *S. pyogenes* NSTIs identified by culture in this study is higher than reported elsewhere, with an accordingly decreased incidence of polymicrobial infections [5, 6, 42, 43], although one patient (patient 6) was found by culture to harbor additional microorganisms. This difference in findings may be due to the relative few samples included in the present study.

### Culture vs. molecular methods

Microorganisms could be identified by the molecular methods in all samples with the exception of samples from patient 10. Thus molecular methods identified microorganisms in cases where no growth was observed by culture. Overall, there were a total of 17 samples where culture and molecular methods were in agreement, giving either concordant (13 samples) or partially concordant results (four samples). The partial concordance is attributable to the greater diversity found by the molecular methods vs. culture (Tables 2 and 3 and Fig. 1), which is consistent with similar comparative studies of other clinical conditions evaluating microbial detection methods [25, 44–48]. In the remaining three cases (4B, 7B and 8C) the disagreement between culture and molecular methods were caused by lack of culturability. Antibiotic therapy may be the cause of this discrepancy, since other samples taken from patients earlier in the course of disease did show growth of microorganisms, which were in agreement with the findings by molecular methods (Table 2). Interestingly, these three cases originated from three different patients where multiple samples were taken and only a single sample gave negative results by culture, illustrating that spatial distribution of pathogenic microorganisms may be an issue that must be taken into account during interpretation of results.

### Molecular methods - agreement to a certain extent

Four different molecular methods were used to identify microorganisms: 1) Microseq (direct Sanger sequencing); 2) 16S rRNA gene clone libraries and Sanger sequencing (except for samples 4A, 6A, 7A, 8A, 8B and 8C due to insufficient amounts of DNA sample); 3) the Ibis T5000 biosensor; and 4) 454-based 16S rRNA gene pyrosequencing. Although all of the molecular methods generally provided concordant results, there were some cases where a microorganism was only detected by one of the four methods (Table 3, Fig. 1). Only microorganisms found by at least two molecular methods were considered to be present in the sample. For a single patient (patient 10) the various molecular methods gave discrepant or negative results. The number of sequence reads obtained by 454-pyrosequencing was very low, and it was not possible to construct clone libraries due to negative PCR for patient 10. These findings indicates that the obtained results (Table 3) may be contaminants or background, which is supported by the fact that neither of the FISH probes used in this study gave a detectable signal for the samples obtained from patient 10.

Discrepancies among the molecular methods were primarily associated with the Microseq method, which misidentified or missed the microorganisms that could be found by the other three molecular methods and (most often) culture. In the Microseq reactions the different DNA strands from different species are competing for the same (and limiting) reagents. The method uses only one primer set in contrast to the redundant strategy of the Ibis T5000 biosensor. Furthermore, the Microseq has a limited resolution compared to clone library where different DNA strands are physically picked out and individually sequenced and the 454-pyrosequencing where different DNA molecules are automatically separated before amplification and sequencing. It is therefore possible that the method is oversensitive toward some species of special interest with high affinity for the primers.

In three cases (3A, 5A and 10A) Microseq identified *S. pyogenes* that were not detected by any other method (except possibly by light microscopy for sample 3A as discussed below). Findings of *S. pyogenes* in NF can only in really rare cases be interpreted as a false positive result. Concerning the relative sensitivity of the techniques, all applied methods in this study have various biases e.g. differential amplification, primer choice. The chromatograms as well as the intensity of the raw files from Microseq are of sufficient quality to deem the results as correct. There are no indications as to the Microseq results being false positives or cross contaminations, however we do acknowledge that this may be the case.

### Microseq quantitative results

Interpretation of quantitative results can reveal some interesting aspects of the NSTIs. By qPCR, the presence of *S. pyogenes* was quantified and related to the total number of bacteria in the sample (estimates of cell numbers based on 16S rRNA gene measurements). When comparing results by qPCR it is important to keep in mind that small variations should not take on assumed relevance, since it has been documented that at best there is a 0.5 log_10_ variance between repeats of the same template concentrations [49]. Based on the qPCR results (Fig. 2), *S. pyogenes* generally appears to be the dominant pathogen when it is present in patients, except samples 6A and 7B, where the total number of bacteria seemed to exceed the number of *S. pyogenes*. These samples contained a number of different species, which supports the findings by qPCR. In the cases where *S. pyogenes* was dominant (and sample 6A), all the applied methods were able to detect and identify the pathogen. For sample 6A, the relative abundance of *S. pyogenes* is around 1-2 % according to 454 pyrosequencing and qPCR (Figs. 1 and 2). It is noteworthy that culture identified such a relatively low abundance species, compared to for instance *Bacteroides fragilis* (detected by both Ibis T5000 biosensor and constituted approximately half of the 454 pyrosequencing reads).

An unexpected finding by qPCR was the presence of *S. pyogenes* in sample 7B (approximately 7000 CFUs/mg sample). The only other method to detect the pathogen was 454 pyrosequencing (Fig. 1), where the species constituted less than 0.01 % of the reads (and is therefore not reported in Table 2). Seen in this light, the number of CFUs/mg sample quantified by qPCR seems relatively high, and indicates the importance of relating qPCR measurements to other data (here both qPCR measurements of other species and broad-range methods). Without this comparison, it would be easy to mistakenly focus on *S. pyogenes*, when all the other molecular methods indicate that *Mycoplasma* species and *Fusobacterium necrophorum* are the problem.

The use of qPCR support our criteria of detection of bacteria by at least two methods, since *S. pyogenes* could not be quantified in samples 3A, 5A, 10A and 10B where Microseq had indicated the presence of the pathogen. This is further strengthened by the fact that it was not possible to visualize *Streptococcaceae* in these samples by FISH, although it is possible that cells are present but not visible due to the high detection limit generally associated with FISH. For the remaining samples where streptococci were detected by multiple methods, it was possible to visualize the organisms by FISH (Fig. 3).

### Visual interpretation of the infected tissue

Compared to the aggressive nature of the infection, the relative low number of bacteria generally detected in the debrided tissue samples is somewhat surprising. We speculate that successful antimicrobial treatment is the cause of this, both by reducing the number of pathogens, but possibly also by rendering the pathogens metabolically inactive, which will impede detection by FISH. This seems plausible since the majority of patients in this study survived the NSTI. We cannot, however, rule out that some pathogens were present in the tissue but not detected due to problems during transport and storage of samples for FISH or because they were present in concentrations below the limit of detection for FISH (Fig. 3-A and -B).

### Microbial findings - the (un)usual suspects

The most common finding by molecular methods was *S. pyogenes* as the sole or dominant pathogen (patients 1, 2, 4 and 9). Some cases of polymicrobial infections were found and included *E. coli*, streptococci and the anaerobes *Bacteroides fragilis, Fusobacterium* spp. and *Clostridium* spp. These findings correspond with bacteria previously reported to be present in polymicrobial NSTIs [13, 42, 50]. Furthermore, fungal NSTI due to *Candida albicans* has also been reported [6, 42]. The detection of *Mycoplasma* spp. in polymicrobial NSTI (patient 7 by all molecular methods), is to the best of our knowledge unique. However, animal studies have shown that ulcerative dermal necrosis can be induced in mice by *Mycoplasma arthritidis* [51]. Mycoplasmas are associated with the mucosa and reside primarily in the respiratory tract and rarely penetrate the submucosa, except in cases of immunosuppression or instrumentation. The lack of cell wall makes the mycoplasmas very sensitive to environmental conditions and isolation of mycoplasmas is complicated due specific nutrient requirements and lack of a single optimal media formulation [52], which may explain why they have not been isolated in NSTI patients before. Interestingly, the localization of infection in patient 7 where mycoplasmas were detected was the neck, but originated and spread from a dental focus, corresponds with the association of mycoplasmas with the respiratory tract.

Infections by species such as *A. baumannii* (patient 3) and *S. pneumoniae* (patient 8) as the sole or dominant species are unusual findings. However, *A. baumanii* is an emergent pathogen and has increasingly been recognized as a prevalent and significant nosocomial pathogen associated with sepsis, wound infections, and pneumonia [53]*. A. baumannii* and other *Acinetobacter* sp. have been described as participants in polymicrobial NSTIs [4, 14, 54] and some reports have identified *A. baumannii* as the sole agent in NSTIs. [53, 55–57]. Light microscopy of the tissue revealed Gram-positive cocci in chains, which may be involved in the initial phases of the infection. The Gram-positive cocci may either be *S. pyogenes* as suggested by Microseq (not confirmed by qPCR) or low abundance staphylococci, which were detected by 454-pyrosequencing. The *A. baumanii* involvement in this case is probably explained by the presence of chronic leg ulcers which could be either colonized or harbor the *A. baumanii* as biofilms deep in the ulcer [36, 58, 59]. *S. pneumoniae,* which was dominant in samples from patient 8, is a widespread pathogen that displays enormous heterogeneity with respect to phenotype and pathogenicity, and has been implicated in community-acquired pneumonia, sinusitis, otitis media, orthopaedic infections and meningitis [44, 60–62]. NSTI due to *S. pneumoniae* is rare and has primarily been reported in cases where patients were immunosuppressed or had other underlying conditions [21, 63–67], which does not correspond to the patient history in this case (Table 1). However, serious infections upon septic spread of the *S. pneumoniae* including to joints and bursas, as in this case, is not unusual.

The realization that many pathogens can cause NSTIs, and that no specific combination of species are found in all cases means that clinicians should be prepared to treat any combination of microbial pathogens [4, 42]. Although appropriate antimicrobial treatment cannot cure NSTI, it can help during the acute phase of the infection [4], which highlights the importance of rapid and comprehensive identification of the pathogens involved.

## Conclusion

In conclusion, the results of this study indicate that molecular diagnostic tools would be suitable supplements for culture, particularly the Ibis technology in order to provide fungal coverage. This would allow for rapid identification of NSTI pathogens and help in cases where culture remains negative or the response to the treatment is not sufficiently satisfying. The much faster turnaround time for the diagnostic molecular methods (particularly the Ibis T5000 biosensor and qPCR) makes the use of these methods attractive for pathogen identification in diseases that have rapid progression such as NSTI, since appropriate initial antibiotic treatment is of pivotal significance. The various new next generation sequencing methods have the ability to generate sequence analysis on complex samples in few hours. Furthermore, rapid accurate diagnostics has the potential to prevent unnecessary changes of the antibiotic treatment if the initiated antibiotic treatment is sufficiently covering the findings. The use of molecular methods may increase the risk of identifying colonizers or contaminants to a higher degree, but this may be an acceptable trade to be able to identify the pathogens in all samples, including samples where routine culture tests did not lead to growth of microorganisms. Identification of microorganisms in patients samples by any method has to be followed by an interpretation of the clinical significance of such finding and this is procedure is always individual.

With easier access to the newer diagnostic techniques due to reduced acquisition costs, easier use, increased knowledge of usefulness and interpretation, increased inclusion of identifying resistance genes and virulence factors these newer diagnostic tools will continue to increase in deployment as an indispensable supplement to more traditional diagnostic methods.

## Abbreviations

CFU: Colony forming units
DAPI: 4’,6-diamidino-2-phenylindole
EtOH: Ethanol
FISH: Fluorescence *in situ* hybridization
MALDI-TOF MS: Matrix-assisted laser desorption-ionization time of flight mass spectroscopy
Mbp: Mega base pairs
NR: Non-redundant
NSTI: Necrotizing soft tissue infections
OTU: Operational taxonomic units
PNA: Peptide nucleic acid
QIIME: Quantitative Insights Into Microbial Ecology
qPCR: Quantitative polymerase chain reaction
RT: Room temperature
